# Liquid biopsy kinetics and detection of ERBB2 amplification/HER2-positivity in refractory hepatocellular carcinoma

**DOI:** 10.1016/j.jlb.2023.100009

**Published:** 2023-09-26

**Authors:** Aaditya Srivant Geddam, Areeb Lutfi, Maaz Khan Afghan, Brian M. Currie, Russell Rosenblatt, Arturo B. Ramirez, Erin G. Bayer, Erika Hissong, Pashtoon Murtaza Kasi

**Affiliations:** a Division of Hematology and Oncology, Weill Cornell Medicine, New York, NY, USA; b Division of Vascular and Interventional Radiology, Weill Cornell Medicine, New York, NY, USA; c Division of Gastroenterology and Hepatology, Weill Cornell Medicine, New York, NY, USA; d RareCyte, Inc, Seattle, WA, 98121, USA; e Department of Pathology and Laboratory Medicine, New York Presbyterian/Weill Cornell Medicine, New York, NY, USA

**Keywords:** ctDNA, Liquid biopsy, Circulating tumor DNA, Circulating tumor cells, CTCs, *ERBB2*, HER2, Hepatocellular carcinoma, Trastuzumab, Pertuzumab, Precision medicine, HCC

## Abstract

Hepatocellular Carcinoma (HCC) is the most common primary liver carcinoma, accounting for 75–85% of all cases. For patients with advanced unresectable and/or metastatic HCC, treatment options primarily include immunotherapy combinations and/or oral tyrosine kinase inhibitors. For patients with alpha-fetoprotein (AFP) levels above 400 ng/ml, ramucirumab, an anti-VEGF agent, is also approved in the second-line setting. However, none of these therapies are based on a specific molecular marker and the role of molecular testing is limited. In fact, HCC is unique in the sense that a biopsy may not be pursued if imaging findings are classic for HCC, in the appropriate clinical context. Herein, we report a case of a patient with metastatic HCC and a huge disease burden where a circulating tumor DNA (ctDNA) liquid biopsy done as part of our standard of care serendipitously revealed a very high *ERBB2* copy number amplification (>78). This was corroborated by HER2 immunohistochemical staining, which demonstrated strong diffuse membranous HER2 3+ expression; tissue next-generation sequencing also identified an *ERBB2* unadjusted copy number gain of 183. Furthermore, we were able to demonstrate the feasibility of using both ctDNA and circulating tumor cells (CTCs) to determine the heterogeneity of HER2 and response to dual HER2 blockade trastuzumab/pertuzumab (MyPathway regimen). In addition to the utility of liquid biopsies increasing opportunities for precision medicine, this n-of-1 precision medicine case illustrates and reiterates the need for comprehensive panel-based NGS testing that employs both DNA/RNA for all patients with advanced or metastatic cancers. It also supports the agnostic potential of *ERBB2*/HER2 as an actionable marker.

## Background

Hepatocellular Carcinoma (HCC) is the most common primary liver carcinoma, accounting for 75–85% of all cases [1]. Nearly 750,000 people globally die from hepatocellular carcinoma (HCC), with most patients diagnosed at a late stage of the disease. Following treatment, recurrence rates range from 50 to 70%, usually within the first two years [2]. In the United States, HCC has been the fastest-rising cause of cancer-related deaths, with incidence rates increasing from 4.4 per 100,000 in 2000 to 6.7 per 100,000 in 2012 and a 4.5% annual increase in HCC mortality from 2000 to 2009 [3]. The risk factors include chronic hepatitis B, hepatitis C, heavy alcohol consumption, and steatotic liver disease [4]. Men are at higher risk of developing HCC than women, with an incidence rate of 6.6 per 100,000 men and 3.4 per 100,000 women in North America [5].

While several molecular deficiencies have been identified in the pathogenesis of HCC, such as DNA methylation alterations, chromosomal instability, immunomodulation, epithelial-to-mesenchymal transition, increased HCC stem cells, and microRNA (miRNA) dysregulation, systemic treatment options are generally not targeted towards one specific marker [6]. For localized HCC, treatment options include locoregional therapies such as surgery, liver transplantation, radiofrequency ablation, *trans*-arterial chemoembolization, and/or radioembolization [3,6]. For patients with advanced unresectable and/or metastatic HCC, treatment options primarily include immunotherapy combinations (e.g. atezolizumab with bevacizumab, or tremelimumab and durvalumab (STRIDE regimen), ipilimumab and nivolumab) and/or oral tyrosine kinase inhibitors (e.g. lenvatinib, sorafenib, cabozantinib, or regorafenib) [7,8]. For patients with alpha-fetoprotein (AFP) levels above 400 ng/ml, ramucirumab, an anti-VEGF agent, is also approved in the second-line setting [9].

However, none of these therapies are based on a specific molecular marker, and the role of molecular testing is limited. In fact, a biopsy may not be considered necessary for diagnosis if the imaging findings are classic for HCC, in the appropriate clinical context. Herein we report a case of a patient with metastatic HCC and large disease burden who was serendipitously found to have a very high *ERBB2* copy number amplification by circulating tumor DNA (ctDNA) liquid biopsy, prompting us to perform confirmational testing and act on this actionable marker.

## Findings

A 49-year-old gentleman with a history of hypertension and hepatitis C virus (HCV), who achieved sustained virologic response 3 years prior, presented to the emergency room for evaluation for abdominal discomfort. A single-phase CT scan showed cirrhosis without a mass and the patient was referred to hepatology for further evaluation. Laboratory tests and serum tumor markers were sent, and the AFP level incidentally returned unmeasurable (>200,000 ng/ml - reference range ≤ 8.0 ng/ml). On further imaging, MRI showed a large confluent/infiltrative mass replacing most of the right hepatic lobe with associated small volume tumor thrombus extending into the right main portal vein. There were numerous additional lesions throughout the liver which were consistent with multifocal disease. In addition, abnormally enlarged upper abdominal and retroperitoneal lymph nodes were noted, consistent with nodal metastases. Multiple subpleural nodules were also seen in the bilateral lung bases, suspicious for metastases. Further findings were suggestive of cirrhosis with evidence of portal hypertension including splenomegaly and mild ascites. Given the classical findings, the patient was diagnosed with metastatic hepatocellular carcinoma. Despite the significant burden of the disease, the patient's liver function test and complete blood count were within normal limits except for a borderline elevated aspartate transaminase level (53 U/L) and alkaline phosphatase (197 U/L).

**Fig. 1:**
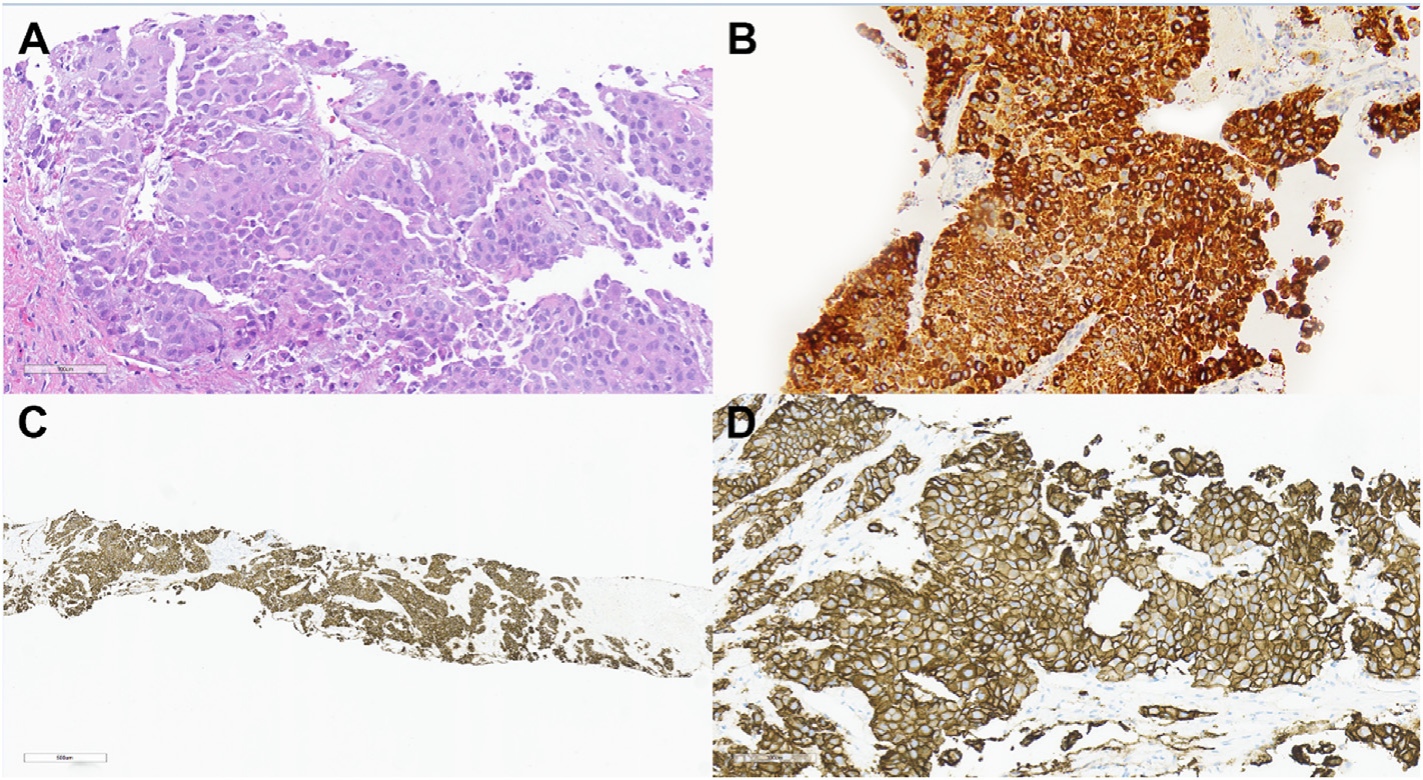
The biopsy demonstrates morphologic features consistent with hepatocellular carcinoma, including nested hepatoid cells with ample eosinophilic cytoplasm. No gland-formation or mucin production was identified (A, 20x). An immunostain for HepPar-1 is diffusely positive in the tumor cells, confirming hepatocyte origin (B, 20x). A low-power view of the HER2 immunostain demonstrates diffuse, strong membranous staining in all tumor cells (C,10x). A higher power view of the HER2 immunostain demonstrates strong, complete membranous staining around tumor cells (D, 20x).

His case was presented at our muti-disciplinary tumor board, and he was started on the newly approved STRIDE regimen; with durvalumab and tremelimumab. Palliative radiation to the dominant mass and tumor thrombus was also recommended, given the liver-dominant nature of his disease. Despite this, the patient rapidly deteriorated clinically with worsening pain and liver function tests in the subsequent weeks. A commercially available liquid biopsy of circulating tumor DNA (ctDNA) (Guardant 360) was performed as part of our routine ‘standard of care’ for all our patients with advanced or metastatic gastrointestinal cancers. Test results reported an *ERBB2* amplification with a very high plasma copy number of 77.5 (reference range, anything above 6–8 is considered high). This was a serendipitous finding. A tissue biopsy pursued in parallel demonstrated infiltration of hepatic parenchyma by a moderately differentiated carcinoma with morphologic features consistent with hepatocellular carcinoma - Fig. 1A. Immunohistochemical stains confirmed hepatocyte origin, with tumor cells showing positivity for Hep-Par1 (Fig. 1B) and arginase, whereas CK7 was negative. Given the ctDNA findings, Human Epidermal Growth Factor Receptor (HER2) expression by immunohistochemistry (IHC) and *ERBB2* amplification by next-generation sequencing on the FFPE biopsy material utilizing a somatic tumor panel (Trusight Oncology 500) were also performed. HER2 immunohistochemistry showed strong and complete membranous staining diffusely throughout the tumor nests (3+) (Fig. 1C and D). Next-generation sequencing on the tissue biopsy revealed a very high focal amplification of *ERBB2*, with 183 copies (unadjusted for tumor content).

**Fig. 2:**
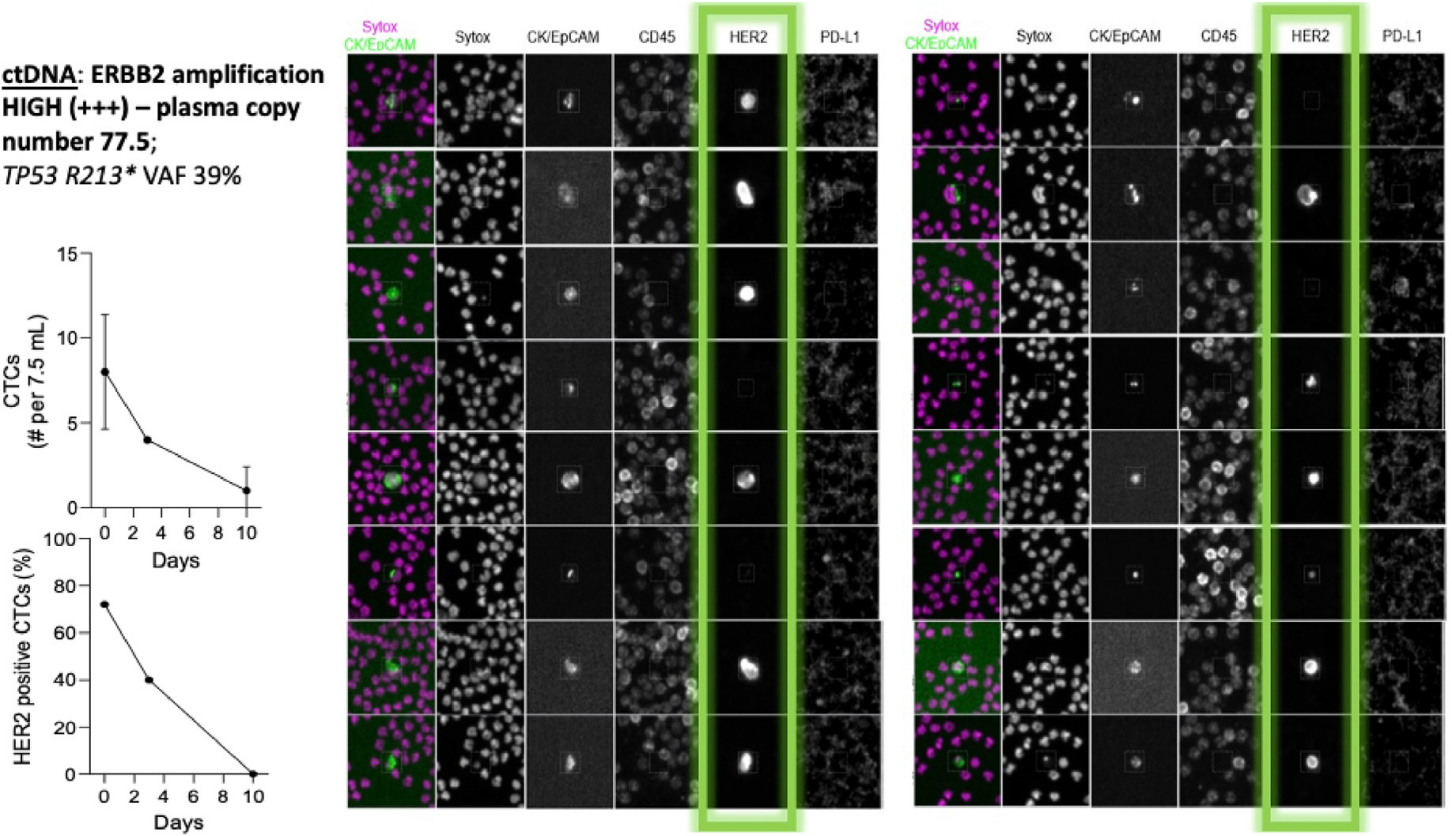
Results of the commercial circulating tumor DNA NGS testing showing the very high *ERBB2* amplification number and other co-mutations/aberrations. Circulating tumor cells (CTCs) were measured using Rarecyte's AccuCyte®, an unbiased density-based method for collecting nucleated cells from whole blood onto slides and staining for CTC markers including HER2 and PDL1 (Day 0: 8 CTCs per 7.5 ml; Day 3: 4 CTCs per 7.5 ml and by Day 10 down to 1 CTC per 7.5 ml). Most cells stained very vividly for the HER2 immunofluorescence stain. However, one-third of the cells are noted to be negative for HER2, reflecting likely tumor heterogeneity. Also, notice the proportion of CTCs that are HER2 positive declining over subsequent timepoints.

Given concerns for rapid clinical progression, we obtained coverage for the dual HER2 blockade trastuzumab/pertuzumab (MyPathway regimen) and were able to show clinical and biomarker response to the actionable target [10]. Furthermore, during the first month of therapy, while the AFP was consistently not measurable (>200,000 ng/ml), the CTCs measured at baseline halved within 3 days of starting the regimen and by Day 10 were down to 1 CTC per 7.5 ml. This was using Rarecyte's AccuCyte®, an unbiased density-based method for collecting nucleated cells from whole blood onto slides and staining for CTC markers including HER2 and PDL1 (Day 0: 8 CTCs per 7.5 ml; Day 3: 4 CTCs per 7.5 ml and by Day 10 down to 1 CTC per 7.5 ml - Fig. 2).

With respect to methodology for liquid biopsy testing and turnaround times, it is important to note that handling and shipping procedures are straightforward. For ctDNA it is standard Streck tubes Cell-Free DNA BCT® and for CTCs it is AccuCyte® Blood Collection Tube (BCT). No special processing or handling is required. Samples can be stored and shipped at room temperature. Testing can be performed up to 14 days for ctDNA collections and for a Clinical Laboratory Improvement Amendments (CLIA) CTC report, ideally within 7–8 days of collection. Turnaround for results has been around 7–9 days for ctDNA commerically availabel platforms and 3–5 days for CTCs, which when compared to tissue genomic testing of 2–3 weeks is fairly remarkable. Each type of genomic testing is looking at the tumor with its own lens. Liquid biopsies are non-invasive and have a faster turnaround than tissue genomic testing. Within liquid biopsies, CTC evaluation and testing provides the fastest real-time assessment. For CTCs here, blood samples were processed with the AccuCyte® blood separation technology (RareCyte®, Seattle, WA). Briefly, 7.5 ml of whole blood is processed using a tube and float system to separate the buffy coat from the red blood cells and plasma. The buffy coat is retrieved and spread as a cell monolayer onto 8 microscope slides. The slides are then stained via immunofluorescence on automated stainers for markers that differentiate CTCs from surrounding white blood cells. Slides are automatically scanned on a CyteFinder® instrument (RareCyte) and image files are analyzed by automated software to identify candidate CTCs for confirmation by a trained reviewer. Identification of CTCs requires positive signal with a nuclear-specific dye, positive staining for Cytokeratin and/or Epithelial Cell Adhesion Molecule (EpCAM) and negative for the white blood cell marker CD45. Additional fluorescent channels are used to quantify expression of phenotypic biomarkers on CTCs; in this case the biomarkers tested were HER2 and PD-L1.

Finally, 4 weeks later, AFP became detectable to 153,000 ng/ml, and 111,914 ng/ml on cycle 2 day 1 of trastuzumab/pertuzumab), with robust clinical improvement and down-trending liver function tests. On cycle 4 Day 1 9 weeks into initiating HER2-blockade, AFP is finally down to less than 1000 ng/ml (999.1 ng/ml). After the completion of just one cycle of therapy, radiographic assessment showed a decrease in the size of the primary tumor in the right hepatic lobe from a maximal length of 16.2 cm–13.6 cm, as well as an overall decrease in the size of multiple other hepatic lesions (e.g., 2.3 cm segment 7 lesion previously was 3.0 cm). No new discrete lesions were identified, and an overall shrinkage was seen in the upper abdominal and retroperitoneal lymph nodes as well. This ongoing response continues to date on subsequent imaging, clinical and biomarker assessment.

## Conclusions

HER2 overexpression as a result of *ERBB2* amplification has been recognized as a driver alteration in multiple cancer types [11]. This is one marker that may potentially soon get agnostic approval like the precedence of agnostic approval of immunotherapy for mismatch repair deficiency and high tumor mutation burden (TMB-High), as well as 2 approved drugs for NTRK-fusion positive cancers. Overexpression results in membranous staining of tumor cells which can be detected by immunohistochemistry, whereas surrounding normal tissue is generally negative [2]. While biliary tract cancers and specifically, extrahepatic cholangiocarcinomas, often harbor overexpression of HER2, and thus have demonstrated response to various anti–HER2 regimens [12], this is an incredibly rare occurrence in HCC. The role of HER2 overexpression in the pathogenesis of HCC is unclear according to available literature. Shi et al. were able to report that HCC patients with HER2 expression had a worse overall survival and the expression pattern was linked to the tumor stage [2]. However, multiple studies have shown that there is no significant association between HER2 overexpression and any clinicopathological parameters [13,17]. Table 1 summarizes the available literature on HCC patients expressing *ERBB2*/HER2. Some studies have suggested a significant correlation between HER2 overexpression and increased aggressiveness of HCC. However, as noted in Table 1, literature on HER2 and HCC is limited, in part since it is not consistently and uniformly assessed, given its rarity.

**Table 1:** Summary of different studies over the years looking at *ERRB2*/HER2 expression in patients with hepatocellular carcinoma (HCC). As noted, the literature is very sparse and methods not uniform. Overall, a rare finding and no data on treatment outcomes has been reported. Abbreviations: NR, not reported.

Study	Year of study	Total patients (n)	HER2 expression n (%)	HER2 ++ (%)	HER2+++ (%)	Findings/Comments
Ji-Hua Shi et al.i [2]	2019	17	14 (82.3%)	NR	NR	HER2 expression linked with tumor stage and overall survival
Z-H Xian et al. [13]	2005	868	62 (7.1%)	2.4%	0.01%	No significant association; HER2 rarely overexpressed in HCC; uncommon event
Tie-Jun Huang et al. [14]	2004	42	9 (21.4%)	NR	NR	HER-2 oncogene amplification specifically examined; correlation seen with age, AFP level and HBV infection
Chiun Hsu et al. [15]	2002	36	1 (2.78%)	2.78%	NR	HER2 overexpression is rare; not predictive of anti-HER-2/neu regulation
Q Su et al. [16]	1995	134	48 (35.8%)	NR	NR	Possible role in HCC pathogenesis; detailed expression data lacking
L Nakopoulou et al. [17]	1994	71	21 (29.5%)	NR	NR	Not involved in the malignant transformation of hepatocytes; rare event

In summary, herein, we demonstrate the feasibility of using ctDNA and circulating tumor cells (CTCs) for both *ERBB2*/HER2 assessment and its kinetics to determine early response to a targeted therapy regimen. We also demonstrate the heterogeneity of circulating tumor cells in circulation that were HER2-positive and how their proportion changed over time on HER2-blockade. Previously, we have shown the feasibility of using ctDNA for patients with HCC to assess potentially actional markers. While liquid biopsies enhance opportunities for precision medicine, this case illustrates and reiterates the need for comprehensive panel based NGS testing for all patients with advanced or metastatic cancers. This precision medicine case (n=1) demonstrates brisk and tremendous response of a therapy in a tumor type not typically harboring this aberration, but given its actionability showing value in its pursuit.

## Funding statement

This research did not receive any external funding from agencies in the public, commercial, or not-for-profit sectors. The ctDNA and tumor next-generation sequencing-based testing was done as part of the routine standard of care. Testing for CTCs was through expanded access made possible through GI research foundation (GIRF) support of RareCyte. Coverage for the dual HER2 regimen was obtained through clinical care/insurance. The tissue biopsy and scans are as per standard clinical protocol. The patient also consented and enrolled in our Universal/Precision Medicine Protocol for biobanking and other biomarker discovery research.

## Declaration of competing interest

The authors declare the following financial interests/personal relationships which may be considered as potential competing interests:

Erin G. Bayer reports a relationship with RareCyte that includes: employment.

Arturo B. Ramirez reports a relationship with RareCyte that includes: employment.

Pashtoon Murtaza Kasi reports a relationship with 10.13039/100004334Merck & Co Inc that includes: consulting or advisory and funding grants. Pashtoon Murtaza Kasi reports a relationship with Agenus Inc that includes: funding grants. Pashtoon Murtaza Kasi reports a relationship with 10.13039/100008272Novartis Pharmaceuticals Corporation that includes: funding grants. Pashtoon Murtaza Kasi reports a relationship with Advanced Accelerator Applications USA Inc that includes: funding grants. Pashtoon Murtaza Kasi reports a relationship with TerSera Therapeutics LLC that includes: funding grants. Pashtoon Murtaza Kasi reports a relationship with 10.13039/100008497Boston Scientific Corp that includes: funding grants. Pashtoon Murtaza Kasi reports a relationship with Elicio Therapeutics Inc that includes: consulting or advisory and equity or stocks. Pashtoon Murtaza Kasi reports a relationship with Guardant Health Inc that includes: consulting or advisory. Pashtoon Murtaza Kasi reports a relationship with Natera, Inc. that includes: consulting or advisory. Pashtoon Murtaza Kasi reports a relationship with Foundation Medicine Inc that includes: consulting or advisory. Pashtoon Murtaza Kasi reports a relationship with Illumina Inc that includes: consulting or advisory. Pashtoon Murtaza Kasi reports a relationship with BostonGene that includes: consulting or advisory. Pashtoon Murtaza Kasi reports a relationship with Tempus that includes: consulting or advisory. Pashtoon Murtaza Kasi reports a relationship with Bayer Corporation that includes: consulting or advisory. Pashtoon Murtaza Kasi reports a relationship with Eli Lilly and Company that includes: consulting or advisory. Pashtoon Murtaza Kasi reports a relationship with Delcath Systems Inc that includes: consulting or advisory. Pashtoon Murtaza Kasi reports a relationship with IPBA that includes: consulting or advisory. Pashtoon Murtaza Kasi reports a relationship with QED Therapeutics that includes: consulting or advisory. Pashtoon Murtaza Kasi reports a relationship with Boston Healthcare Associates Inc that includes: consulting or advisory. Pashtoon Murtaza Kasi reports a relationship with Servier that includes: consulting or advisory. Pashtoon Murtaza Kasi reports a relationship with Taiho Oncology Inc that includes: consulting or advisory. Pashtoon Murtaza Kasi reports a relationship with Exact Sciences Corporation that includes: consulting or advisory. Pashtoon Murtaza Kasi reports a relationship with Daiichi Sankyo Inc that includes: consulting or advisory. Pashtoon Murtaza Kasi reports a relationship with Eisai Inc that includes: consulting or advisory. Pashtoon Murtaza Kasi reports a relationship with AstraZeneca Pharmaceuticals LP that includes: consulting or advisory and travel reimbursement. Pashtoon Murtaza Kasi reports a relationship with Saga Diagnostics that includes: consulting or advisory. Pashtoon Murtaza Kasi reports a relationship with NeoGenomics Laboratories Inc that includes: consulting or advisory. Pashtoon Murtaza Kasi reports a relationship with Do More Diagnostics that includes: consulting or advisory. Pashtoon Murtaza Kasi reports a relationship with Seagen Inc that includes: consulting or advisory.
